# ODAD4-Related Primary Ciliary Dyskinesia: Report of Five Cases and a Founder Variant in Quebec

**DOI:** 10.3390/cells14181460

**Published:** 2025-09-18

**Authors:** Marie-Hélène Bourassa, Guillaume Sillon, Shuizi Ding, Maurizio Chioccioli, Monkol Lek, Kaiyue Ma, Alejandro Mejia-Garcia, Simon Gravel, Donald C. Vinh, Michael R. Knowles, Margaret W. Leigh, Stephanie D. Davis, Thomas Ferkol, Kenneth N. Olivier, Elizabeth N. Schecterman, Weining Yin, Patrick R. Sears, Martina Gentzsch, Susan E. Boyles, William D. Bennett, Kirby L. Zeman, Lawrence E. Ostrowski, Maimoona A. Zariwala, Adam J. Shapiro

**Affiliations:** 1 Department of Pediatrics, McGill University, Montreal, QC H4A 3J1, Canada; marie-helene.bourassa@mail.mcgill.ca; 2 Division of Medical Genetics, Department of Medicine, McGill University Health Center, Montreal, QC H4A 0B1, Canada; guillaume.sillon@mcgill.ca; 3 Department of Human Genetics, McGill University, Montreal, QC H4A 3J1, Canada; alejandro.mejiagarcia@mail.mcgill.ca (A.M.-G.); simon.gravel@mcgill.ca (S.G.); 4 Section of Pulmonary, Critical Care and Sleep Medicine, Yale University School of Medicine, New Haven, CT 06510, USA; dingshuizi@csu.edu.cn (S.D.); maurizio.chioccioli@yale.edu (M.C.); 5 Department of Genetics, Yale University School of Medicine, New Haven, CT 06510, USA; monkol.lek@yale.edu (M.L.); kaiyue.ma@aya.yale.edu (K.M.); 6 Division of Infectious Diseases, Department of Medicine, Research Institute of the McGill University Health Centre, Montreal, QC H4A 3J1, Canada; donald.vinh@mcgill.ca; 7 Department of Medicine, School of Medicine, University of North Carolina, Chapel Hill, NC 27599, USA; michael_knowles@med.unc.edu (M.R.K.); kenneth_olivier@med.unc.edu (K.N.O.); 8 Marsico Lung Institute, Cystic Fibrosis Research Center, University of North Carolina, Chapel Hill, NC 27599, USA; weining_yin@med.unc.edu (W.Y.); patrick_sears@med.unc.edu (P.R.S.); martina_gentzsch@med.unc.edu (M.G.); susan_burkett@med.unc.edu (S.E.B.); ostro@med.unc.edu (L.E.O.); maimoona_zariwala@med.unc.edu (M.A.Z.); 9 Department of Pediatrics, School of Medicine, University of North Carolina, Chapel Hill, NC 27599, USA; margaret_leigh@med.unc.edu (M.W.L.); stephanie_davis@med.unc.edu (S.D.D.); ferkol@email.unc.edu (T.F.); elizabeth_schecterman@med.unc.edu (E.N.S.); 10 Center for Environmental Medicine, Asthma, and Lung Biology, University of North Carolina, Chapel Hill, NC 27599, USA; william_bennett@med.unc.edu (W.D.B.); kirby_zeman@med.unc.edu (K.L.Z.); 11 Department of Pediatrics, Research Institute of the McGill University Health Centre, Montreal, QC H4A 3J1, Canada

**Keywords:** primary ciliary dyskinesia, *ODAD4*, founder variant, mild phenotype, Quebec

## Abstract

Pathogenic variants in *ODAD4* are an ultra-rare cause of primary ciliary dyskinesia (PCD). Previously reported cases display classic disease phenotypes, including chronic oto-sino-pulmonary disease and development of bronchiectasis by adulthood. We report five individuals with PCD harboring biallelic *ODAD4* variants (median age 14, range 3–41 years). Participants underwent standardized PCD diagnostic evaluations. Three individuals shared the novel homozygous *ODAD4* genotype [NM_031421.5: c.245delA, p.(Lys82Argfs*29)], and genealogy analysis highly suggests a founder effect in French-Canadians from two regions of Quebec. All five participants had normal pulmonary function values. Two Quebec participants lacked radiographic pneumonias or bronchiectasis (ages 14 and 38 years) despite life-long suppurative respiratory symptoms, low nasal nitric oxide levels, and outer dynein arm defects on electron microscopy. Reverse transcription polymerase chain reaction of the c.245delA variant showed abnormal splicing with in-frame skipping of exon 2, allowing expression of a mildly shortened mRNA product. However, functional analysis showed overall static cilia, absence of ODAD4 protein on Western blot, and absence of in vivo mucociliary clearance. The reason for a milder pulmonary phenotype with the c.245delA variant in *ODAD4* remains unclear, but regional screening for this variant in Quebec may identify more cases and enhance understanding of this mild form of PCD.

## 1. Introduction

Primary ciliary dyskinesia (PCD, OMIM 244400) is a genetically and clinically heterogeneous disorder of motile cilia, leading to suppurative sino-oto-pulmonary disease. Characterized by defects in ciliary ultrastructure, function, or biogenesis, PCD results in impaired mucociliary clearance, recurrent lower respiratory tract infections, and eventually bronchiectasis in nearly all adult patients [1,2,3,4]. Other clinical manifestations of PCD include neonatal respiratory distress, chronic rhinosinusitis, recurrent otitis media, subfertility, and organ laterality defects (e.g., *situs inversus totalis* or *situs ambiguus*) [5,6,7,8]. Diagnosis of PCD is often challenging as there is no single diagnostic test that detects all forms, and many of the recommended diagnostic modalities, such as nasal nitric oxide measurement (nNO), transmission electron microscopy analysis of ciliary ultrastructure (TEM), and ciliary high-speed video microscopy analysis (HSVA), may not be readily accessible depending on location [9,10,11,12,13]. Genetic testing now plays an important role in the diagnosis of PCD, a primarily autosomal recessive disease caused by variants in 1 of more than 55 different genes [14,15,16]. With the ever-increasing number of causative PCD genes identified, variants in ultra-rare PCD genes remain poorly characterized and lack extensive description of genotype–phenotype correlations [17].

Loss of the outer dynein arm (ODA) is the most common ultrastructural defect seen in PCD, and this defect may be caused by variants in more than 10 distinct ODA genes [18,19,20,21]. Disease-causing variants in the *ODAD4* gene (also known as *TTC25*, located on chromosome 17q21.2, OMIM 617095) are extremely rare and affect the ODA docking complex (ODA-DC), therefore impeding attachment of the ODAs to the doublet microtubules, resulting in nearly complete ciliary immotility [22,23,24]. *ODAD4*-associated PCD has been reported in 19 individuals thus far; all of the described cases presented with typical respiratory phenotypes and some, including affected children, with documented bronchiectasis (Table S4) [3,24,25,26,27,28,29]. Here, we describe five unrelated (no known familial relations) cases of PCD with disease-causing variants in *ODAD4*, including three cases from Quebec harboring identical variants through a likely founder effect with an unusually mild respiratory phenotype.

## 2. Materials and Methods

### 2.1. Subjects

Individuals with *ODAD4*-related PCD were identified from research protocols at the McGill University Centre (MUHC) in Montreal, Quebec, Canada, and the Genetic Disorders of Mucociliary Clearance Consortium (GDMCC), with 8 North American research locations led by the University of North Carolina (UNC). All participants signed informed consent, and protocols were approved by the local institutional review boards of the MUHC or the UNC.

### 2.2. Diagnostic Testing and Genetic Analysis

All patients were referred for clinical suspicion of PCD. Past radiology, respiratory microbiology, clinical laboratory testing, and PCD diagnostic testing were collected. Per previously published protocols, the patients underwent standardized evaluations using approved methodology, including history and physical examination, nNO measurement, ciliary TEM, and next-generation sequencing (NGS) genetic panels testing of >40 PCD-associated genes [30,31]. With inconclusive genetic panels, two patients (QC-01 and QC-03) were investigated by whole exome sequencing (WES) by research laboratories at the MUHC. Briefly, MUHC patients had repatriation of their WES backbone data from a commercial laboratory (Blueprint Genetics, Seattle, WA, USA) for further in-house bioinformatics analysis, followed by targeted variant confirmation at Blueprint Genetics. One GDMCC participant from Quebec (QC-02) with an inconclusive NGS panel underwent targeted *ODAD4* variant analysis by Sanger sequencing. Two other GDMCC participants (NC-01 and NC-02) from outside of Quebec underwent WES by the Yale Center for Genome Analysis, followed by variant annotation and filtration strategies described earlier [32]. Variant pathogenicity was assigned per the American College of Medical Genetics and Genomics and the Association for Molecular Pathology variant classification standards [33].

### 2.3. Nasal Epithelial Cell Analysis

Two participants (QC-01, QC-02) underwent detailed analysis of nasal epithelial samples. For this, nasal epithelial cells were scraped from the inferior turbinate using a Rhinoprobe device without topical anesthesia and examined for ciliary activity. The cells were then analyzed in 1 of 2 different laboratories at UNC (QC-01) or Yale University (QC-02) after regrowth at air–liquid interface (ALI) [32]. HSVA of the epithelial cells was performed pre- and post-ALI essentially as previously described [32]. Bioelectric properties of the cultured cells from QC-01 were examined using Ussing chambers as previously described [34].

The cultured nasal cells from QC-02 underwent reverse transcription polymerase chain reaction (RT-PCR) analysis at Yale University and were compared to cells from a healthy control. Briefly, RNA was extracted using the AllPrep DNA/RNA Micro Kit (QIAGEN, 80204, Venlo, The Netherlands) and treated with the ezDNase Enzyme (Invitrogen, 11766051, Thermo Fisher Scientific, Waltham, MA, USA). Reverse transcription was performed using 1 µg ezDNase-treated RNA and the PrimeScript RT-PCR Kit (TaKaRa, RR014, Shiga, Japan). PCR was performed using cDNA made from 200 ng RNA and the Q5 High-Fidelity DNA Polymerase (NEB, M0491, Ipswich, MA, USA). Using the NucleoSpin Gel and PCR Clean-Up kit (TaKaRa; 740609, Shiga, Japan), RT-PCR products were gel purified for Sanger sequencing and column purified for Amplicon-EZ NGS (Genewiz, South Plainfield, NJ, USA). The primer sequences are provided in Supplementary Materials (Figure S3). Western blot analysis for ODAD4 proteins was performed on QC-01 and compared against cells from a healthy control, as previously described [35]. The antibodies used are listed in Supplementary Materials Table S2.

### 2.4. In Vivo Mucociliary Clearance Measurement (MCC)

Mucociliary clearance testing was performed on QC-01 at UNC per standardized protocol and compared to an average of 8 previously studied healthy controls [36]. Briefly, subjects inhaled a tracer compound (Tc99m-labeled sulfur colloid), and retention of the labeled particles was measured using a planar gamma camera. The percentage of clearance is calculated as [(1 − retention) × 100]. Baseline MCC was measured for 60 min, followed by measuring albuterol-stimulated MCC for 60 min. Cough clearance was measured by having the subject perform a series of 30 coughs over the next 30 min.

### 2.5. Analysis of a Quebec Founder Variant

To investigate a possible common ancestor for carriers of the *ODAD4* variant c.245delA in Quebec, we accessed whole genome sequencing (WGS, n = 2173) and genotype array data (n = 29,337) from the CARTaGENE cohort, which includes genotype and health data on 43,000 individuals aged 40–69 years in Quebec [37]. From the genotype data, we reconstructed the ancestral recombination graph (ARG) using ARG-needle (https://palamaralab.github.io/software/argneedle/, accessed on 17 July 2025) [38]. We first identified carriers using WGS and estimated their time to the most recent common ancestor (TMRCA). We then used *ODAD4* variant c.245delA carriers identified by WGS to perform genotype imputation with the ARG, as described previously [39]. We selected ARG-imputation as it outperforms other standard imputation methods for ultra-rare variants (MAF < 0.01%) [38]. In addition, we compared ARG-imputation against standard imputation using the TOPMED imputation server (panel TOPMed-r2, n = 97,256 deeply sequenced human genomes as reference). We kept individuals who were imputed by both strategies for downstream analysis.

Imputed CARTaGENE carriers who provided consent were linked to the BALSAC database, a genealogy catalog constructed from over 400 years of historical birth, marriage, and death records since the initial European (France) settlement of Quebec. We also linked our three reported patients with homozygous c.245delA variants to the BALSAC database. Next, we simulated the allele transmission of the *ODAD4* variant c.245delA using ISGen software (https://github.com/DomNelson/ISGen, accessed on 17 July 2025) to identify the most likely founders responsible for this introduction [40]. ISGen performs allele climbing simulations to identify likely founders and estimates regional frequencies conditional on these simulated ancestries. After founder identification, allele frequency estimation was performed with ISGen for 24 historic regions in Quebec. We performed 100,000 climbing simulations. Confidence intervals for allele frequencies were calculated using 100,000 bootstrap iterations by resampling individuals within each region.

## 3. Results

We identified five unrelated individuals who were referred for suspicion of PCD, with bi-allelic variants in *ODAD4*. Patient ages at diagnosis were 41 (QC-01), 11 (QC-02), 3 (QC-03), 3 (NC-01), and 10 (NC-02) years, with an average age at diagnosis of 14 years. Only one patient was diagnosed in adulthood. Three participants were White and of French-Canadian descent (QC-01, QC-02, QC-03), while the two other participants (NC-01, White; NC-02, Hispanic) were from the United States. The three French-Canadian individuals all shared the same genotype with homozygous *ODAD4* [NM_031421.5, c.245delA, p.Lys82Argfs*29] variants, while the non-French-Canadian participants had compound heterozygous variants, one (NC-01) including the c.245delA variant along with c.731delC, p.(Pro244Argfs*11), and another (NC-02) with variants at c.100C>T, p.(Gln34*) and c.1145 + G>A (splice donor) (Table 1, Figure S1). Through parental genetic testing, biallelic segregation was confirmed in two cases. One parent was available for testing in the other three cases, which showed they only carried one variant, highly suggesting that the proband variants arise *in trans* (Figure S1). The homozygous c.245delA variant has not been previously published in PCD cases, but the variant is found in heterozygosity in 121 of all 1,613,730 alleles in gnomAD v.4.1. This frameshift variant in exon 2 (of 12 exons total) is predicted to result in a premature stop codon, thereby leading to a loss of protein expression, either through protein truncation or nonsense-mediated mRNA decay, and therefore, loss of function. This variant has been reported in ClinVar (variation ID 869383) and classified as pathogenic.

**Figure 1:**
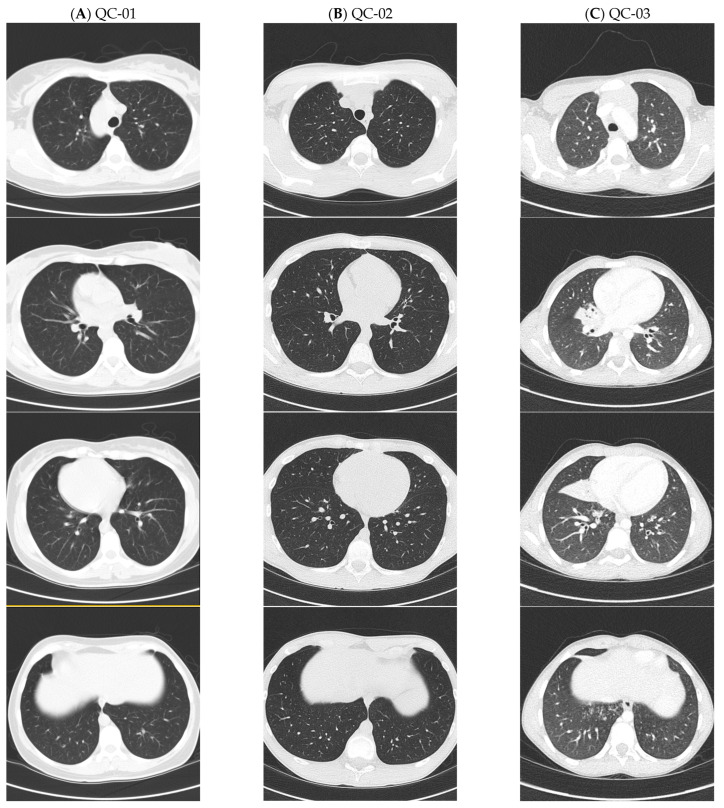
Lung CT scan images from patients with *ODAD4*-related PCD. Caption: (**A**) normal lung CT images with absence of bronchiectasis in QC-01 at 38 years old, (**B**) normal lung CT images with absence of bronchiectasis in QC-02 at 14 years old, and (**C**) right middle lobe atelectasis with bronchiectasis seen on lung CT images of QC-03 at 8 years old.

All five participants presented with typical PCD symptoms, including year-round wet cough and year-round nasal congestion present since birth (Table 2). Pulmonary function values were normal in all participants, with a forced expiratory volume in 1 s (FEV1) ranging from 89 to 107% predicted. Laterality defects were noted in all but one patient, and two had a history of neonatal respiratory distress. Most patients had severe sinus disease, which led to functional endoscopic sinus surgery in two cases, with one having confirmed nasal polyposis. Four participants had recurrent otitis media requiring multiple tympanostomies, and three had hearing loss (two mild, one severe), which continued into adulthood for the single adult patient who has ongoing severe hearing loss requiring hearing aids. This same patient (QC-01) failed one attempt of intrauterine insemination but eventually had two spontaneous pregnancies after 5 years. Despite an otherwise classic otolaryngologic PCD phenotype with situs inversus totalis, this adult patient had mild lower respiratory disease, lacking recurrent pneumonias on chest radiography or bronchiectasis on chest computed tomography (CT) at 38 years of age (Figure 1). Another French-Canadian patient (QC-02) also lacked pneumonias on chest radiography and bronchiectasis on chest CT scan at 14 years old. However, participant QC-03, with the identical c.245delA homozygous variant, had recurrent pneumonias and right middle lobe bronchiectasis on CT scan at 8 years of age. Recurrent pneumonias and bronchiectasis were reported in NC-01, who underwent lobectomy at 4 years old, while NC-02 never had pneumonias on chest radiography but did not undergo chest CT scanning to look for bronchiectasis. Sputum microbiology cultures in all cases (obtained while patients were in their baseline states of health) showed organisms commonly seen in patients with PCD, and more pathogenic organisms, like *Pseudomonas aeruginosa*, were isolated in three patients (Table 2).

**Figure 2:**
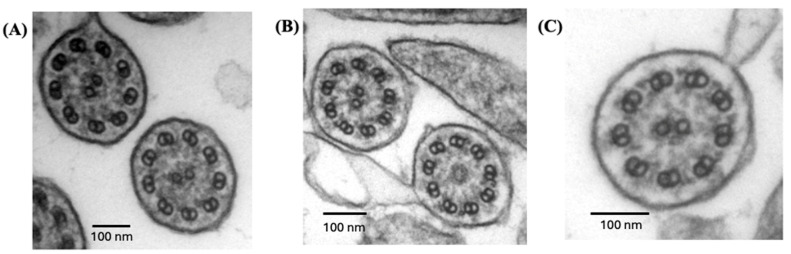
Ciliary transmission electron microscopy of patients QC-01 and QC-02 with *ODAD4* variants. Nasal ciliary electron microscopy images showing (**A**) QC-01 with complete absence of ODA in 92% of cross-sections, (**B**) QC-01 with occasional full-length ODA seen in 8% of all ciliary cross-sections, and (**C**) QC-02 with full-length ODA seen in 15% of all ciliary cross-sections.

All individuals had low nNO values (<77 nL/min) on at least one occasion. Transmission electron microscopy showed absent ODAs in >80% of all ciliary cross-sections, consistent with previously reported cases in the literature [24,26,27,42]. However, 8–15% of ciliary cross-sections had identifiable ODAs on a few peripheral microtubules in patients from Quebec (Table 1, Figure 2).

**Figure 3:**
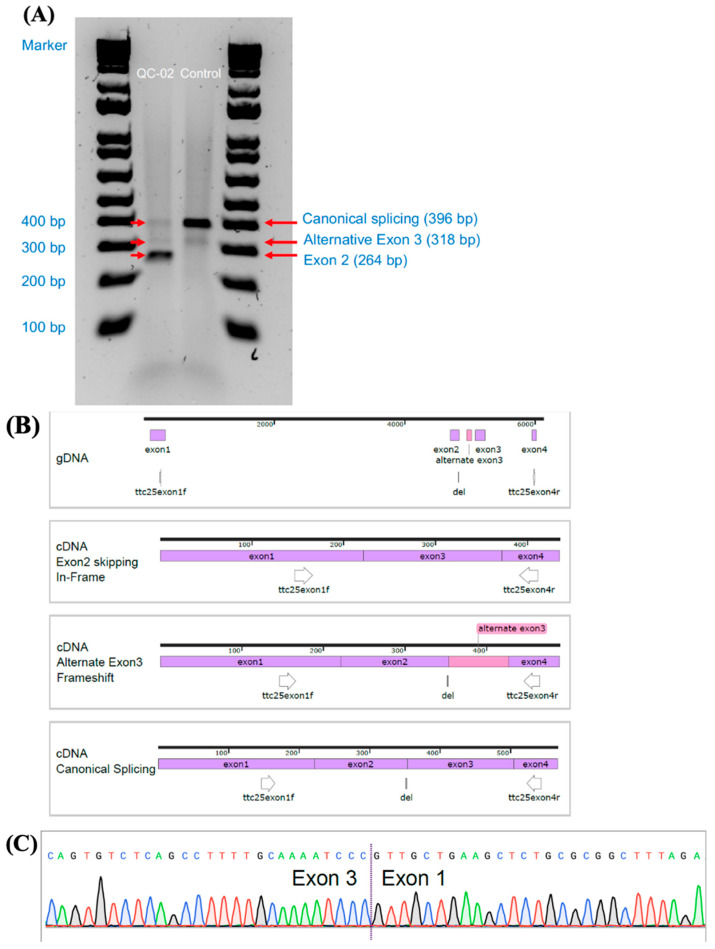
RT-PCR analysis of nasal epithelial cell cultures. Caption: RT-PCR analysis of nasal epithelial cell cultures of patient QC-02 showing an in-frame splice site defect. (**A**) RT-PCR gel electrophoresis using RNA from nasal epithelial cells of QC-02 with homozygous c.245delA variants and a healthy control (canonical transcript: ENST00000377540; transcript with alternative exon 3: ENST00000593239). (**B**) Schematic diagram of the splicing events and placement of primers for RT-PCR of ODAD4 c.245delA −/− cells. (**C**) Electropherogram of exon-2-skipped transcript sequence in QC-02 (The reverse primer was used for Sanger sequencing). An alternate exon 3 was seen in 6% of transcripts from QC-02, but it was also observed in the healthy control sample and was considered insignificant.

Because subjects QC-01 and QC-02 with homozygous c.245delA exhibited a mild respiratory phenotype, we performed additional analysis on these participants. Direct examination of ciliated cells from a nasal scrape showed overall absent movement with only occasional uncoordinated twitching of cilia (Video S1). Similarly, HSVA of cells after differentiation in an air–liquid interface showed overall static cilia, without any significant ciliary activity (Videos S2 and S3), including eight evenly spaced fields from nine different cultures for a total of 72 videos for QC-01. Additionally, several differentiated cultures were incubated at 30 degrees Celsius for up to 7 days in attempts to stabilize the truncated ODAD4 protein, but no increase in ciliary activity was detected. The RT-PCR analysis of nasal epithelial cells from patient QC-02 showed abnormal splicing with in-frame skipping of exon 2 (out of 12 coding exons), which was confirmed by Sanger sequencing and next-generation sequencing (Figure 3). This led to a shortened product of 264 base pairs (bp) in 91% of the RNA transcripts instead of the 396 bp product seen in the healthy control. An alternate exon 3 was seen in 6% of transcripts, which was also observed in the healthy control sample and was considered insignificant. The remainder of RT-PCR transcripts in QC-02 showed greatly reduced canonical splicing (3%) compared to the healthy control (Figures S2 and S3, Table S1).

To investigate ODAD4 expression at the protein level, a Western blot analysis was performed on lysates from differentiated cultures of nasal epithelial cells from subject QC-01. Immunostaining with polyclonal antibodies targeting the N-terminal region did not detect full-length wild-type protein (76.6 kD; Figure S4A). Because the antibodies targeted a sequence including exon 2, which is predicted to be deleted in the majority of the RNA transcripts, we also used polyclonal antibodies targeting the C-terminal region of ODAD4. While ODAD4 was readily detected in lysates from the healthy controls, no protein (including a truncated one of predicted size 71.8 kD) was detected in the QC-01 sample, even though the axonemal protein (RSPH1) was detected at similar levels, indicating approximately equal loading of ciliary proteins (Figure S4B).

To assess airway epithelial ion transport, which is essential for maintaining proper mucus hydration to support effective cough and mucociliary clearance, electrophysiological analyses were conducted in Ussing chambers on nasal epithelial cultures from QC-01 differentiated at the air–liquid interface [34]. Under these conditions, cystic fibrosis transmembrane conductance regulator (CFTR), epithelial sodium channel (ENaC), and calcium-activated chloride channel (CaCC) activity were all within the normal range (Figure S5).

Finally, we studied whole lung clearance of radiolabeled tracer particles in patient QC-01 for evidence of residual mucociliary clearance (Figure S6). The results showed negligible clearance: baseline average percentage clearance from 0 to 60 min was 0.08% and albuterol-stimulated clearance from 60 to 120 min was 0.3% compared to values of 7.7% and 9.2% in a previously studied group of healthy controls (n = 8). The absence of detectable MCC is consistent with the observed lack of ciliary activity. Cough clearance was also reduced compared to healthy controls (healthy controls, 10.5%; QC-01, 4.6%).

**Figure 4:**
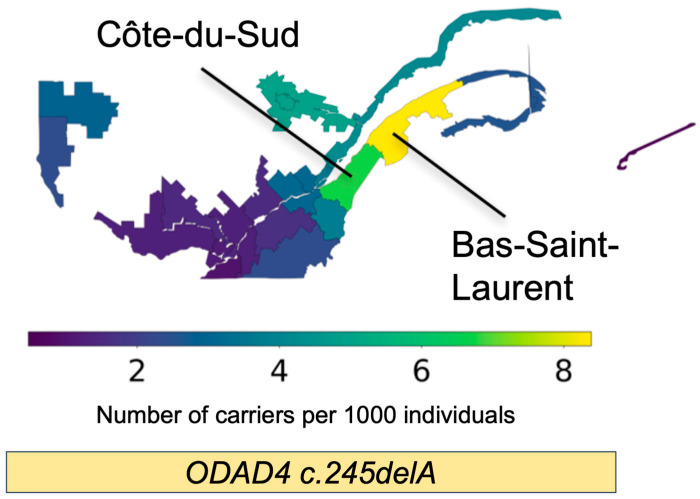
Regional frequency estimation of the *ODAD4* c.245delA variant in Quebec using ISGen. Quebec heatmap showing the carrier frequency across 24 historical regions of Quebec, as defined by the BALSAC project. The *ODAD4* c.245delA variant displays a higher frequency in the Bas-Saint-Laurent and Côte-du-Sud regions of Quebec.

In the Quebec genealogy analysis, we identified three carriers of c.245delA in CARTaGENE using WGS. The estimated TMRCA between carriers was 11.40 generations (330 years ago). ARG-imputation identified 59 imputed carriers, of which 20 consented and had enough information for linkage to the BALSAC demographics database (Table 3). The TOPMED Imputation Server identified 67 carriers. We kept 59 imputed carriers identified by both imputation approaches for downstream analysis. TMRCA is inversely correlated with haplotype length, demonstrating that all the imputed carriers shared a haplotype containing this variant. Finally, regional frequency estimation across Quebec showed a higher carrier frequency in two specific, neighboring regions of Quebec: Bas-Saint-Laurent (1/111) and Côte-du-Sud (1/143), which both follow the southern bank of the Saint Lawrence River in Eastern Quebec (Figure 4, Table S3).

## 4. Discussion

This report presents five cases of ODAD4-related PCD, including novel pathogenic genetic variants, and significantly expands the number of PCD cases with ODAD4 variants in the published literature [3,24,25,26,27,28,29]. ODAD4 encodes a 672 amino acid protein, which contributes to docking the ODA on peripheral microtubules along the entire length of the ciliary axoneme [24]. Past reports of ODAD4-related PCD show absent ODA on TEM, low nNO values, and largely immotile cilia on HSVA, which agree with our findings in this report [24,25,26,27]. Patients with PCD from ODA defects and associated genes also present with a classic PCD phenotype, including recurrent lower respiratory tract infections with pneumonias, obstructive lung disease with impaired pulmonary function over time, and development of bronchiectasis in nearly all patients by adulthood. Situs anomalies were quite prevalent in our cohort, noted in 80% of patients. A high prevalence of situs defects is reported in PCD with variants in other ODA-docking complex genes [43,44], though some specific docking complex genes have also been associated with lower amounts of situs anomalies [45]. Thus, it is unclear if the higher prevalence of situs anomalies in ODAD4-related PCD is due to a more critical role in nodal cilia function compared to other ODA-docking complex genes or simply due to referral bias. We identified a novel, homozygous c.245delA variant in ODAD4 associated with a mild respiratory phenotype in two cases, including lack of pneumonias on chest radiography, relative preservation of pulmonary function values, and absence of bronchiectasis in an adult patient who was diagnosed with PCD in the fourth decade of life. However, our youngest patient with homozygous c.245delA variants actually had features of a more classic pulmonary phenotype, including right middle lobe bronchiectasis on chest CT scan at age 8.

The c.245delA variant was identified in four of our *ODAD4* cases, in homozygous arrangement in three French-Canadian individuals from Quebec and in compound heterozygous arrangement with a different pathogenic variant in another participant without known French-Canadian heritage. The c.245delA variant was presumed to cause a frameshift variant in exon 2, resulting in a premature stop codon and loss of normal protein function. However, RT-PCR analysis showed an aberrant transcript causing in-frame skipping of exon 2, potentially allowing for the expression of a shortened protein (44 amino acids shorter than wild type). With this finding, we hypothesized that partial/residual protein function explained the milder phenotype seen in some patients with c.245delA variants. This was supported by the intermittent ODA presence seen on 8–15% of TEM cross-sections and the milder pulmonary phenotypes of the two oldest PCD patients. However, the largely immobile cilia on HSVA refute the notion of residual ciliary activity as the cause of a mild phenotype. This was further countered by the absence of detectable ODAD4 protein on the Western blots of epithelial cells and the lack of in vivo mucociliary clearance in our adult participant with homozygous c.245delA variants.

The reason for mild respiratory phenotypes in our two oldest participants remains unclear. Neither had significant pulmonary function impairment, yet both still suffer from chronic suppurative lung disease with daily cough productive of sputum. Exploration of possible compensatory mechanisms in respiratory ENaC and CFTR channels did not show altered function to support their protective involvement. Past studies have shown milder respiratory phenotypes with variants in other PCD genes, including *DNAH11* and *RSPH*1 [35,46], but not in other genes causing ODA defects. However, variability in PCD phenotype may also occur across variants within the same gene depending on the type of sequence alteration that occurs (loss of function versus residual function), and this has been reported in both human and murine models [47,48]. Genetic modifiers outside of the disease-causing gene alter phenotypes in other suppurative respiratory conditions (cystic fibrosis), but these have not been investigated in PCD cohorts [49]. In cystic fibrosis, genotype-focused modulator therapies significantly improve lower respiratory disease [50,51,52,53]. It is similarly conceivable that genetic modifier therapies may eventually improve ciliary function in PCD, but further research is needed in this domain. Discovering the pathophysiologic mechanisms underlying milder forms of PCD, as we introduced in this report, will be critical to the development of therapies that may lessen the long-term respiratory deterioration in this condition.

Our genealogy analysis of the c.245delA variant suggests that it may contribute to PCD cases across Quebec, and more specifically to cases concentrated in the Bas-Saint-Laurent and Côte-du-Sud regions. This is consistent with the well-documented founder event that occurred in Bas-Saint-Laurent after the arrival of French individuals to Quebec in the 1600s [54]. ARG and genealogical analyses also strongly suggest that the c.245delA variant is a founder variant in Quebec, with a single founder introduction. As the pulmonary phenotype associated with the c.245delA variant may be mild in some cases, and the extent of variable expressivity is unknown, it is possible that people with this variant are not clinically considered for PCD and seldom investigated for this condition. Programs targeting patients with bronchiectasis for PCD genetic testing in these higher burden regions of Quebec will be necessary to understand the true prevalence of *ODAD4*-related PCD.

## 5. Conclusions

Disease-causing variants in ODAD4 are a rare cause of PCD, usually resulting in classic phenotypes with lower respiratory tract infections, progressive obstructive lung disease, and bronchiectasis. The c.234delA variant in ODAD4 seems to arise from a founder effect in French-Canadians from Quebec, and in some cases, produces a mild lower respiratory phenotype without signs of bronchiectasis or pulmonary function impairment in adulthood. While the mechanisms underlying this milder phenotype remain unclear, increased recognition of phenotype variability will be essential to detect, diagnose, and treat future patients with PCD due to variants in ODAD4.

## Figures and Tables

**Table 1:** Demographics and diagnostic tests in participants with *ODAD4* variants.

Patient ID	Gender	Age at Diagnosis (Years)	Ethnicity	*ODAD4* (TTC25) Variants	nNO (nL/Min)	**TEM**	**HSVA**
QC-01	F	41	White, French-Canadian	c.245delA, p.(Lys82Argfs*29)—Path, HOM	10, 12	Absent ODA 92% of sections	No activity
QC-02	M	11	White, French-Canadian	c.245delA, p.(Lys82Argfs*29)—Path, HOM	13, 6, 21	Absent ODA 85% of sections	No activity
QC-03	M	3	White, French-Canadian	c.245delA, p.(Lys82Argfs*29)—Path, HOM	6 *	Absent ODA 100% of sections	ND
NC-01	M	3	White	c.245delA, p.(Lys82Argfs*29)—Path; c.731delC, p.(Pro244Argfs*11)—Path, Compd Het	15	Absent ODA 100% of sections	ND
NC-02	M	10	Hispanic	c.100C>T, p.(Gln34*)–Path; c.1145 + 1G>A, p.(Splice donor)—Path, Compd Het	26	Absent ODA 91% of sections	ND

Compd Het—compound heterozygous; F—female; HOM—homozygous; HSVA—high speed videomicroscopy analysis; M—male; ODA—outer dynein arm; Path—pathogenic; ND—not done; nNO—nasal nitric oxide; TEM—transmission electron microscopy. * nNO via tidal breathing at 3 years old. Exhalation against resistor technique is used for all other nNO measurements in cases when ≥5 years. Some participants had multiple nNO measurements performed on separate visits. Normal nNO levels are 304.6 +/− 118.8 nL/min (mean +/− SD), calculated from 78 healthy subjects [41].

**Table 2:** Clinical symptoms and results of surveillance PCD testing.

Patient	NRDS	Chronic Rhino-Sinusitis	ROM	Chronic Wet Cough	Recurrent Pneumonia	Bronchiectasis on CT, Lobe Affected (Age Detected)	Situs	FEV1 (%)	Sputum Organisms
QC-01	No	Yes	Yes	Yes	No	No (38)	SIT	95	OP flora, MBKub
QC-02	Yes	Yes	Yes	Yes	No	No (14)	SS	107	StAur, MCat, StrPne, PsA (eradicated)
QC-03	Yes	No	Yes	Yes	Yes	Yes—RML (8)	SA	106	StAur, StrPyo MCat, HFlu
NC-01	No	Yes	Yes	Yes	Yes	Yes—RLL (4)	SIT	89	StAur, Hflu, MCat, StrPne, PsA
NC-02	No	Yes	No	Yes	No	CT not done	SIT	100	PsA

CT—computed tomography; FEV1—forced expiratory volume in 1 s; HFlu—*Haemophilus influenzae*; MCat—*Moraxella catarrhalis*; MBKub—*Mycobacterium kubicae*; NRDS—neonatal respiratory distress syndrome; OP—oropharyngeal; PsA—*Pseudomonas aeruginosa*; ROM—recurrent otitis media; RML—right middle lobe; RLL—right lower lobe; SA—situs ambiguus; SIT—situs inversus totalis; SS—situs solitus; StAur—*Staphylococcus aureus*; StrPne—*Streptococcus pneumoniae*; StrPyo—*Streptococcus pyogenes*.

**Table 3:** c.245delA founder pathogenic variant causing PCD in Quebec.

			CARTaGENE				
GRCh38 Genomic Location	TMRCA (Generations)	GnomAD NFE	WGS	ARG Imputation	TOPMED Imputation	Bas Saint- Laurent	Côte du Sud
ODAD4 (TTC25): chr17-41935345-TA-T	11.4	1/5840	3	59	67	1/111	1/143

ARG—ancestral recombination graph; TMRCA—time to the most recent common ancestor. GnomAD NFE—carrier frequency in the GnomAD database for Europeans (Non-Finnish). WGS—whole genome sequencing.

## Data Availability

The original contributions presented in this study are included in the article/Supplementary Materials. Further inquiries can be directed to the corresponding author(s).

## Supplementary Materials

The following supporting information can be downloaded at https://www.mdpi.com/article/10.3390/cells14181460/s1. Table S1—Sanger sequencing validation of the different RNA transcripts in QC-02. Table S2—Antibodies used for Western blots. Table S3—Carrier frequency estimation in Regions of Quebec using ISGen. Table S4—Summary of *ODAD4 (TTC25)* (NM_031421.5) cases harboring pathogenic variants in previously published and current reports. Figure S1—Pedigree and genomic sequences showing segregation analysis and location of pathogenic variants in *ODAD4* (TTC25) (NM_031421.5) in 5 unrelated kindreds. Figure S2—Relative abundance of mapped Amplicon-EZ reads from different transcripts in RT-PCR analysis of patient QC-02. Figure S3—Protocol and primer sequences used for genotyping and RT-PCR analysis in participant QC-02 at Yale University. Figure S4—Western blot analysis for ODAD4 protein in nasal epithelial cells of participant QC-01. Figure S5—In vitro bioelectric measurements of nasal epithelial cultures from QC-01. Figure S6—In vivo lung clearance testing in participant QC-01. Video S1—High-speed video microscopy analysis on freshly sampled nasal epithelial cells in participant QC-01. Video S2—High-speed video microscopy analysis of nasal epithelial cells in participant QC-02 after regrowth at the air–liquid interface. Video S3—High speed video microscopy analysis of nasal epithelial cells from participant QC-01 after regrowth at air-liquid interface [2,24,25,26,27,28,29,34,36,42].
