# Supplementary material for: ODAD4-Related Primary Ciliary Dyskinesia: Report of Five Cases and a Founder Variant in Quebec
Source: Cells. 2025 Sep 18;14(18):1460. doi: 10.3390/cells14181460 (PMC12468610; doi:10.3390/cells14181460)
Supplement: Supplementary file 1 [file cells-14-01460-s001.zip › Supplementary Materials.pdf]

Supplemental Information

Video S1: High speed video microscopy analysis on freshly sampled nasal epithelial cells in participant QC-01.

Caption: The sample was placed in a glass chamber in an environmental chamber (OkoLab) set at 37°C with 5% CO2 humidified air. Cells were inspected visually and videos from areas with cilia (n=13) were recorded using a Nikon Eclipse TE2000 inverted microscope using a 60x oil objective (NA=1.4) with DIC optics and 2x post-objective magnification. Videos were recorded at 120 frames per second with a Basler acA1300 camera controlled by SAVA software (Ammons Engineering, Cilo, MI, USA). Only occasional, abnormal, uncoordinated twitching motion was observed; video shows 3 examples of areas with the highest motility.

Video S2: High speed video microscopy analysis of nasal epithelial cells in participant QC-02 after regrowth at air-liquid interface.

Caption: Video showing overall lack of ciliary movement with occasional flickering of isolated cilia.

Video S3: High speed video microscopy analysis of nasal epithelial cells from participant QC-01 after regrowth at air-liquid interface.

Caption: Here are 3 representative areas from a total of 18 videos (n= 3 cultures) are shown. None of the cultures examined demonstrated any measurable ciliary activity.

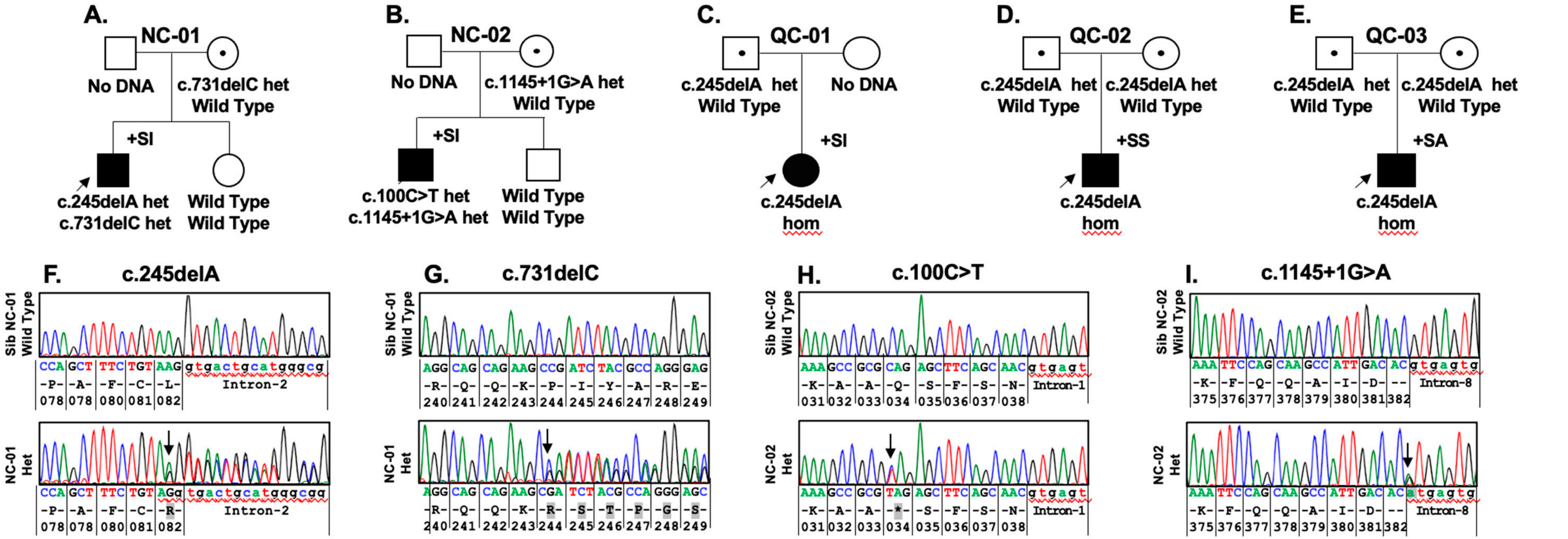

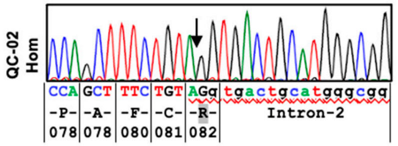

Figure S1: Pedigree and genomic sequences showing segregation analysis and location of pathogenic variants in *ODAD4 (TTC25)* (NM_031421.5) in 5 unrelated kindreds.

A: Whole exome sequencing identified two pathogenic variants [c.245delA, p.(Lys82Argfs*29), Exon 2] and [c.731delC, p.(Pro244Argfs*11), Exon 6] in *ODAD4* in a proband NC-01.

B: Whole exome sequencing identified two pathogenic variants [c.100C>T, p.(Gln34*), Exon 1] and [c.1145+1G>A, p.(splice?), Intron 8] in *ODAD4* in a proband NC-02.

C-E: Targeted testing (QC-2) and BluePrint panel testing (QC-01 and QC-03) identified homozygous pathogenic variants [c.245delA, p.(Lys82Argfs*29), Exon 2] in *ODAD4* in probands.

F-I: Representative electropherograms showing the locations of the pathogenic variants. Base sequences, amino acid sequences and codon numbers are indicated. Location of the mutation is shown by an arrow, and highlighted grey for the base or amino acid location.

Males and females are designated by the squares and circles, respectively. Filled symbol and symbols with a dot within show affected and carrier individuals, respectively. Proband is designated with an arrow next to the filled symbol. Situs solitus (SS), Situs inversus (SI), Situs ambiguous (SA), homozygous (HOM), and heterozygous (het) are labeled.

Figure S2: Relative abundance of mapped Amplicon-EZ reads from different transcripts in RT-PCR analysis of patient QC-02.

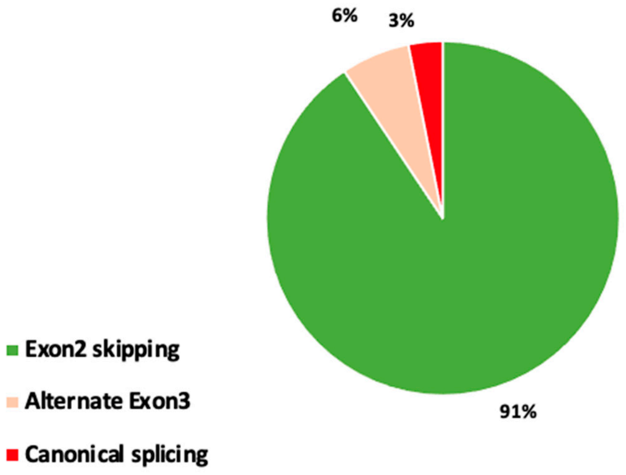

Caption: The RT-PCR product was column purified and sent for the Amplicon-EZ Next-generation sequencing. Read counts were quantified using unique sequences assigned to these 3 transcripts exclusively (grep <unique_seq> R1 | wc -l)+ (grep <unique_seq> R2 | wc -l).

Table S1 - Sanger sequencing validation of the different RNA transcripts in QC-02.

Band	Size (bp)	Annotation	Transcript	Frame	Sanger sequencing
1	264	Exon2 skipping	ENST00000377540	In-Frame	Validated
2	318	Alternate Exon3	ENST00000593239	Frameshift	Not validated (too noisy)
3	396	Canonical splicing	ENST00000377540	Frameshift	Validated (poor quality)

Caption: RT-PCR bands were gel purified and sent for Sanger sequencing. The band resulting from in-frame exon 2 skipping was confirmed by Sanger sequencing (Figure 3C). The other two bands were too faint to be explicitly validated using Sanger sequencing. The presence of all 3 transcripts was validated using Amplicon-EZ NGS (Figure S2).

Figure S3. Protocol and primer sequences used for genotyping and RT-PCR analysis in participant QC-02 at Yale University.

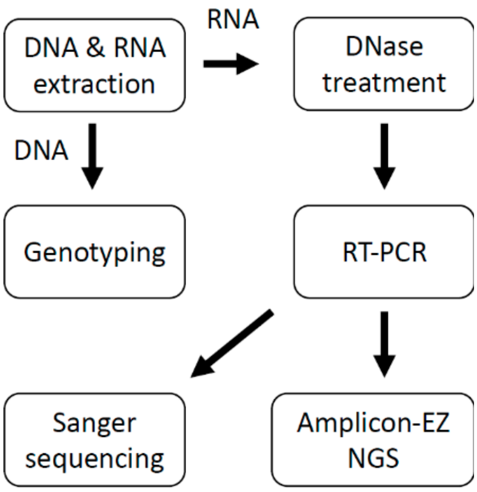

Caption: Step 1: DNA and RNA extraction were performed using AllPrep DNA/RNA Micro Kit (QIAGEN; 80204). Step 2: Genotyping PCR was performed using Q5 High-Fidelity DNA Polymerase (NEB, M0491). The following primers were used. F: GACAAGAACTGCCTGGTTGC (5'->3'); R: TCCCACTGAGTTGTTGATGG (5'->3'). Step 3: The RNA samples were treated with ezDNase™ Enzyme (Invitrogen; 11766051). Step 4: cDNA synthesis was performed using PrimeScript RT-PCR Kit (TaKaRa; RR014). Q5 High-Fidelity DNA Polymerase and the following primers were used for the PCR. Exon1_F: CCCTCTTATATGGCCGAAGG (5'->3'); Exon4_R: AGAGGTCCCCTTTGTTCTCC (5'->3'). Step 5: RT-PCR product was gel purified and sent for Sanger sequencing and column purified for Amplicon-EZ NGS (Genewiz).

Figure S4: Western blot analysis for *ODAD4* protein in nasal epithelial cells of participant QC-01.

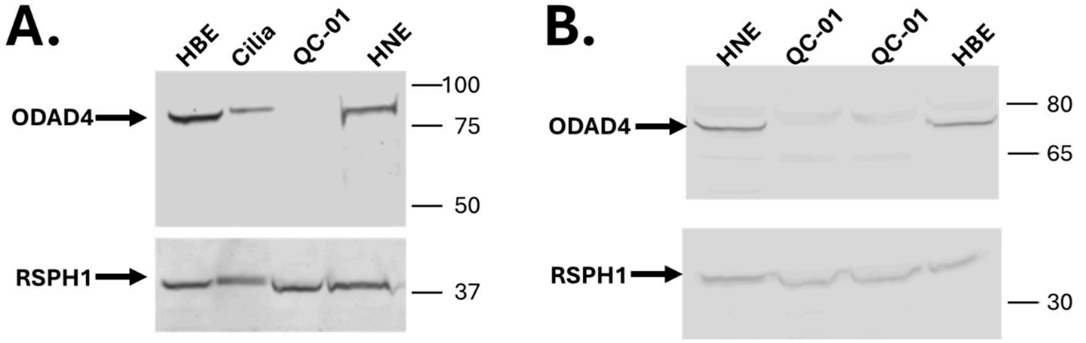

Caption: Western blot analysis of cultured nasal epithelial cells from subject QC-01 with an antibody against A) the N-terminal region of ODAD4 and B) an antibody against the C-terminal region of ODAD4. ODAD4 protein is clearly present in the control samples, but no protein is detectable in the QC-01 lysate. Samples were also probed with an antibody against RSPH1 to demonstrate similar levels of ciliary protein loading.
HBE, cultured human bronchial epithelial cells from healthy control; cilia, isolated human cilia; QC-01, cultured nasal epithelial cells from subject QC-01; HNE, cultured nasal epithelial cells from healthy control.

Table S2: Antibodies used for Western blots.

Protein	Antigen	Type	Dilution	Source
ODAD4	N-terminal	Rabbit polyclonal	1:2000	Atlas Antibodies HPA023908
ODAD4	C-terminal peptide	Rabbit polyclonal	1:500	Invitrogen PA5-70945
RSPH1	C-terminal peptide	Rabbit polyclonal	1:2000	Atlas Antibodies HPA016816

Figure S5: In vitro bioelectric measurements of nasal epithelial cultures from QC-01.

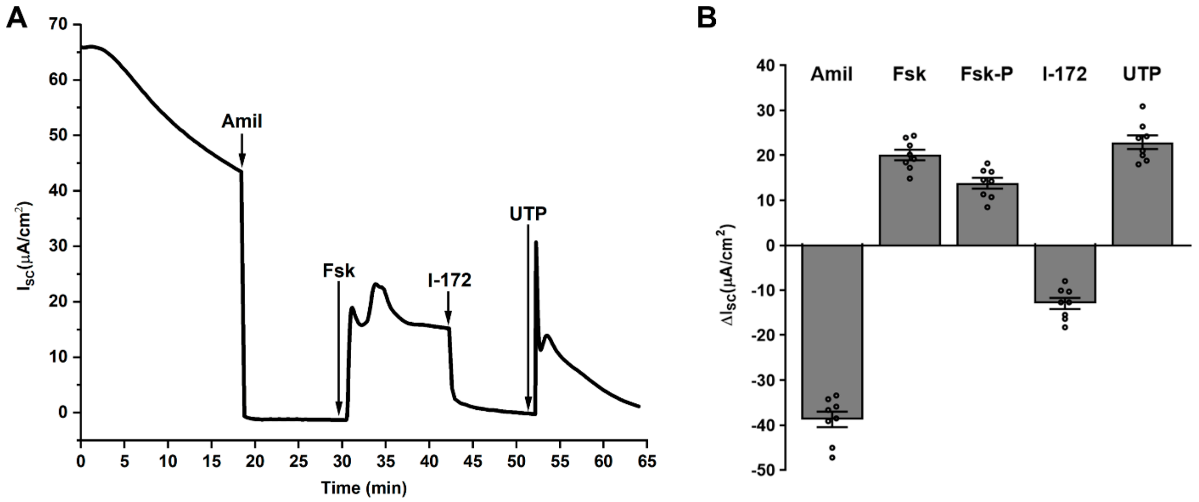

Caption: Bioelectric properties of differentiated air-liquid interface cultures of nasal epithelial cells from QC-01 were measured in Ussing chambers as previously described[1]. **A.** Representative short-circuit current (I_{sc}) trace measured in Ussing chambers in response to various sequentially added agonists and antagonists. **B.** Quantitation of responses to amiloride (Amil) to assess ENaC function, forskolin (Fsk) to activate CFTR (Fsk=maximal response, Fsk-P = forskolin plateau), CFTR inhibitor (I-172) to inhibit CFTR function, and UTP to measure CaCC activity were recorded. All responses were within the normal range (Mean ± SEM, n = 8).

Figure S6: In vivo lung clearance testing in participant QC-01.

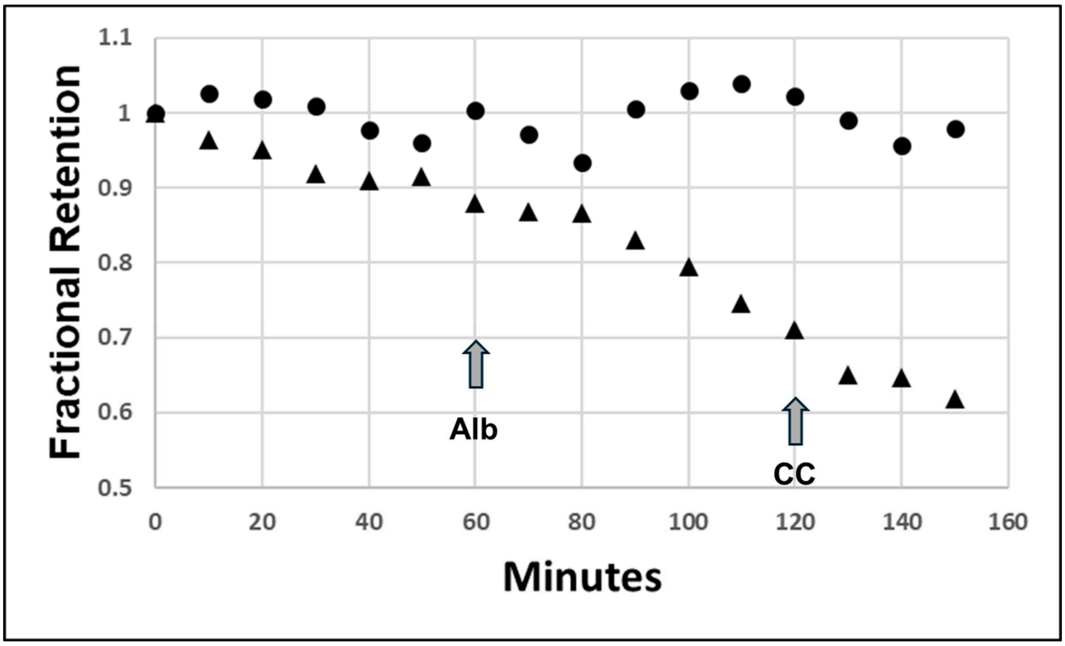

Caption: Whole lung clearance was measured in subject QC-01 (circles) and compared to a previously studied group of healthy controls (n=8; diamonds). Each subject inhaled a radioactive tracer at time=0 and the clearance of the tracer from the lungs was monitored over time by gamma scintigraphy following an established protocol[2]. Baseline MCC was measured from 0-60 minutes, albuterol stimulated MCC (Alb) was measured from 60-120 minutes, and clearance by controlled cough (CC) was measured from 120-150 minutes. At t=150 minutes, healthy controls show an average retention of 0.6, indicating clearance of ~40% of the deposited tracer, while clearance in subject QC-01 was negligible. See text for further details.

Table S3: Carrier frequency estimation of *ODAD4* c.245delA in regions of Quebec using ISGen.*

Region	Estimated Carrier Frequency (per 1000 individuals)**	95% Confidence Interval	Sample size	Carrier Prevalence
Ile-de-Montréal	1.41	1.35-1.45	1235	1/737
Saguenay-Lac-St-Jean	4.92	4.35-5.92	486	1/230
Charlevoix	4.23	3.55-5.46	94	1/282
Lanaudière	1.29	1.18-1.36	241	1/846
Estrie	2.39	2.30-2.50	442	1/435
Québec	3.07	3.03-3.13	354	1/331
Rive-Sud-de-Montréal	0.97	0.95-0.99	155	1/1055
Lévis-Lotbinière	2.39	2.30-2.51	212	1/434
Beauce	3.81	3.51-4.18	264	1/285
Rive-Nord-Ouest-de-Montréal	1.19	1.09-1.26	170	1/918
Côte-Nord	4.12	3.86-4.43	43	1/259
Richelieu	1.26	1.17-1.34	278	1/856
Laurentides	1.49	1.31-1.63	147	1/764
Côte-de-Beaupré	3.63	3.29-4.17	52	1/304
Témiscamingue	2.37	2.27-2.46	23	1/440
Iles-de-la-Madeleine	0.47	0.41-0.55	30	1/2415
Abitibi	2.92	2.84-3.04	31	1/353
Outaouais	1.29	1.18-1.39	180	1/847
Gaspésie	2.60	2.40-2.77	178	1/416
Mauricie	1.51	1.47-1.56	391	1/681
Bois-Francs	1.64	1.56-1.71	444	1/640
Portneuf	1.67	1.61-1.74	130	1/623
**Côte-du-Sud**	**6.77**	**6.31-7.15**	**325**	**1/159**
**Bas-Saint-Laurent**	**8.39**	**7.76-8.82**	**359**	**1/129**

*Refer to methods section for further details.

95% confidence interval was computed using bootstrapping with 100,000 iterations.

**Carrier frequency is computed by allele dropping simulations within the BALSAC genealogy. The resulting sample size reflects the number of present-day individuals per region in the genealogy.

Table S4: Summary of *ODAD4*/TTC25 (NM_031421.5) cases harboring pathogenic variants in previously published and current report.

Study	Case/family Id	Age (yrs)	Genomic variant, Zygosity	Protein variant	Variant type	Parental Consanguinity	nNO nl/min	Laterality defect	ROM	NRDS	Bronchiectasis	TEM	HSVA
Wallmeier et al., 2016[3]	OP-95 II2	NA	c.114+1G >T, Hom	P?	Canonical Splice Donor	Y	LOW	Y	NA	N	Y	Absent ODA	NA
Wallmeier et al., 2016[3]	OP-1331 II3	NA	c.425_426insT, Hom	p.Lys142Asnfs*1	Frameshift	Y	LOW	Y	NA	Y	Y	Absent ODA	NA
Wallmeier et al., 2016[3]	OP-1331 II1	NA	c.425_426insT, Hom	p.Lys142Asnfs*1	Frameshift	Y	LOW	N	NA	N	Y	Absent ODA	NA
Mani et al., 2019[4]	GM01590/17GM01727	3	c.655_657delinsTGTGGAGGACCTCATCCTCC	p.(Leu219Cysfs*14)	Frameshift	NA	NA	Y	N	N	NA	NA	NA
Mani et al., 2019[4]	DC2224/16GM00070	6	c.655_657delinsTGTGGAGGACCTCATCCTCC	p.(Leu219Cysfs*14)	Frameshift	Y	NA	Y	Y	N	NA	NA	NA
Mani et al., 2019[4]	DC2224/DCP1867	41	c.655_657delinsTGTGGAGGACCTCATCCTCC	p.(Leu219Cysfs*14)	Frameshift	NA	NA	N	Y	N	Y	NA	NA
Emiralioğlu et al., 2020[5]	Case #8	10	c.716G>A	p.Trp239*	Nonsense	Y	NA	N	N	Y	Y	NA	Stiff pattern
Backman et al., 2021[6]	Single case	<1	c.1145+1G >A, appears Hem	p.?	Canonical Splice Donor	N	NA	Y	Y	Y	NA	Absent ODA	NA
Backman et al., 2021[6]	Single case	<1	50-kb deletion, Het	(arr[GRCh37] 17q21.2(40077958_40128088)x1)	Gross deletion	N	NA	Y	Y	Y	NA	Absent ODA	NA

Yiallouros et al., 2021[7]	0AVBQLMH	AVG 30	c.716G>A	p.Trp239*	Nonsense	N	LOW	N	Y	N	NA	ODA+IDA*	Immotile/almost immotile
Yiallouros et al., 2021[7]	V00D410F		c.716G>A	p.Trp239*	Nonsense	N	LOW	Y	N	Y	NA	ODA+IDA*	Immotile/almost immotile
Yiallouros et al., 2021[7]	B0786QT8		c.716G>A	p.Trp239*	Nonsense	N	LOW	Y	N	Y	NA	ODA+IDA*	Immotile/almost immotile
Zlotina et al., 2024[8]	Case 17 (II 1)	17	c.704dup, Hom	p.His235Glnfs*48	Frameshift	Y	NA	NA	NA	NA	Y	NA	Totally immotile cilia
Raidt et al., 2025[9]	na	na	c.245delA, Het	p.(Lys82Argfs*29)	Frameshift**	NA	NA	NA	NA	NA	NA	NA	NA
Raidt et al., 2025[9]	na	na	c.397+1G>A, Zygosity?	p.?	Canonical Splice Donor	NA	NA	NA	NA	NA	NA	NA	NA
Raidt et al., 2025[9]	na	na	c.451C>T, Zygosity? unknown	p.(Gln151*)	Nonsense	NA	NA	NA	NA	NA	NA	NA	NA
Raidt et al., 2025[9]	na	na	c.619del, Het	p.(Asp207Metfs*4)	Frameshift	NA	NA	NA	NA	NA	NA	NA	NA
Raidt et al., 2025[9]	na	na	c.716G>A, Zygosity unknown?	p.(Trp239*)	Nonsense	NA	NA	NA	NA	NA	NA	NA	NA
Raidt et al., 2025[9]	na	na	Microduplication 17q21.2, Het	p.?	Microduplication	NA	NA	NA	NA	NA	NA	NA	NA
Current study	QC-1	41	c.245delA, Hom	p.(Lys82Argfs*29)	Frameshift	N	LOW	Y	Y	N	N	Absent ODA	Immotile
Current study	QC-02	11	c.245delA, Hom	p.(Lys82Argfs*29)	Frameshift	N	LOW	N	Y	Y	N	Absent ODA	Immotile
Current study	QC-03	3	c.245delA, Hom	p.(Lys82Argfs*29)	Frameshift	N	LOW	Y	Y	Y	Y	Absent ODA	NA
Current study	NC-01	1 mo	c.245delA, Het	p.(Lys82Argfs*29)	Frameshift	N	LOW	Y	Y	N	Y	Absent ODA	NA

Current study	NC-01	1 mo	c.731delC, Het	p.(Pro244Argfs*11)	Frameshift	N	LOW	Y	Y	N	Y	Absent ODA	NA
Current study	NC-02	5	c.100C>T, Het	p.(Gln34*)	Nonsense	N	LOW	Y	N	N	NA	Absent ODA	NA
Current study	NC-02	5	c.1145+1G>A, Het	p.?	Canonical Splice Donor	N	LOW	Y	N	N	NA	Absent ODA	NA

Het - Heterozygous, Hem - Hemizygous, Hom – Homozygous, AVG – average, HSVA - high-speed video microscopy analysis, NA – not available, nNO – Nasal nitric oxide value, NRDS neonatal respiratory distress syndrome, ROM – recurrent otitis media, TEM - transmission electron microscopy, Yrs – years, ODA – outer dynein arm, IDA – inner dynein arm.

* Usually, ODA defects are seen. IDA defects can be non-specific[10].

** Variant resided near splice donor site and mRNA analysis in current study revealed an in-frame skipping of exon 2 consisting of 44 amino acid residues.

References

1. McCravy MS, Quinney NL, Cholon DM, Boyles SE, Jensen TJ, Aleksandrov AA, et al. Personalised medicine for non-classic cystic fibrosis resulting from rare CFTR mutations. Eur Respir J. 2020;56(1). doi:10.1183/13993003.00062-2020.
2. Bennett WD, Wu J, Fuller F, Balcazar JR, Zeman KL, Duckworth H, et al. Duration of action of hypertonic saline on mucociliary clearance in the normal lung. J Appl Physiol (1985). 2015;118(12):1483-90. doi:10.1152/japplphysiol.00404.2014.
3. Wallmeier J, Shiratori H, Dougherty GW, Edelbusch C, Hjeij R, Loges NT, et al. TTC25 Deficiency Results in Defects of the Outer Dynein Arm Docking Machinery and Primary Ciliary Dyskinesia with Left-Right Body Asymmetry Randomization. Am J Hum Genet. 2016;99(2):460-9. doi:10.1016/j.ajhg.2016.06.014.
4. Mani R, Belkacem S, Soua Z, Chantot S, Montantin G, Tissier S, et al. Primary ciliary dyskinesia gene contribution in Tunisia: Identification of a major Mediterranean allele. Hum Mutat. 2020;41(1):115-21. doi:10.1002/humu.23905.
5. Emiralioglu N, Taskiran EZ, Kosukcu C, Bilgic E, Atilla P, Kaya B, et al. Genotype and phenotype evaluation of patients with primary ciliary dyskinesia: First results from Turkey. Pediatr Pulmonol. 2020;55(2):383-93. doi:10.1002/ppul.24583.
6. Backman K, Mears WE, Waheeb A, Beaulieu Bergeron M, McClintock J, de Nanassy J, et al. A splice site and copy number variant responsible for TTC25-related primary ciliary dyskinesia. Eur J Med Genet. 2021;64(5):104193. doi:10.1016/j.ejmg.2021.104193.
7. Yiallouros PK, Kouis P, Kyriacou K, Evriviadou A, Anagnostopoulou P, Matthaiou A, et al. Implementation of multigene panel NGS diagnosis in the national primary ciliary dyskinesia cohort of Cyprus: An island with a high disease prevalence. Hum Mutat. 2021;42(6):e62-e77. doi:10.1002/humu.24196.
8. Zlotina A, Barashkova S, Zhuk S, Skitchenko R, Usoltsev D, Sokolnikova P, et al. Characterization of pathogenic genetic variants in Russian patients with primary ciliary dyskinesia using gene panel sequencing and transcript analysis. Orphanet J Rare Dis. 2024;19(1):310. doi:10.1186/s13023-024-03318-3.
9. Raidt J, Riepenhausen S, Pennekamp P, Olbrich H, Amirav I, Athanazio RA, et al. Analyses of 1236 genotyped primary ciliary dyskinesia individuals identify regional clusters of distinct DNA variants and significant genotype-phenotype correlations. Eur Respir J. 2024;64(2). doi:10.1183/13993003.01769-2023.
10. O'Callaghan C, Rutman A, Williams GM, Hirst RA. Inner dynein arm defects causing primary ciliary dyskinesia: repeat testing required. Eur Respir J. 2011;38(3):603-7. doi:10.1183/09031936.00108410.
